# Prevalence and incidence of low back pain among runners: a systematic review

**DOI:** 10.1186/s12891-020-03357-4

**Published:** 2020-06-03

**Authors:** Filippo Maselli, Lorenzo Storari, Valerio Barbari, Andrea Colombi, Andrea Turolla, Silvia Gianola, Giacomo Rossettini, Marco Testa

**Affiliations:** 1 Department of Neurosciences, Rehabilitation, Ophthalmology, Genetic and Maternal Infantile Sciences (DINOGMI), University of Genova - Campus of Savona, Savona, Italy; 2 Sovrintendenza Sanitaria Regionale Puglia INAIL, Bari, Italy; 3 IRCCS, San Camillo, Laboratory of Rehabilitation Technologies, Rehabilitation Research Unit, Venice, Italy; 4 IRCCS, Istituto Ortopedico Galeazzi, Unit of Clinical Epidemiology, Milano, Italy

**Keywords:** Low Back pain, incidence, Prevalence, Running

## Abstract

**Background:**

Running is one of the most popular sports worldwide. Despite low back pain (LBP) represents the most common musculoskeletal disorder in population and in sports, there is currently sparse evidence about prevalence, incidence and risk factors for LBP among runners. The aims of this systematic review were to investigate among runners: prevalence and incidence of LBP and specific risk factors for the onset of LBP.

**Methods:**

A systematic review has been conducted according to the guidelines of the PRISMA statement. The research was conducted in the following databases from their inception to 31st of July 2019: PubMed; CINAHL; Google Scholar; Ovid; PsycINFO; PSYNDEX; Embase; SPORTDiscus; Scientific Electronic Library Online; Cochrane Library and Web of Science. The checklists of The Joanna Briggs Institute Critical Appraisal tools were used to investigate the risk of bias of the included studies.

**Results:**

Nineteen studies were included and the interrater agreement for full-text selection was good (K = 0.78; 0.61–0.80 IC 95%). Overall, low values of prevalence (0.7–20.2%) and incidence (0.3–22%) of LBP among runners were reported. Most reported risk factors were: running for more than 6 years; body mass index > 24; higher physical height; not performing traditional aerobics activity weekly; restricted range of motion of hip flexion; difference between leg-length; poor hamstrings and back flexibility.

Conclusions: Prevalence and incidence of LBP among runners are low compared to the others running related injuries and to general, or specific population of athletes. View the low level of incidence and prevalence of LBP, running could be interpreted as a protective factor against the onset of LBP.

**Systematic review registration:**

PROSPERO CRD42018102001.

## Keypoints

- Prevalence and incidence of LBP among runners seem basically low if compared with general population and other popular sports activities;
- Running could, cautiously, be considered a protective factor for the lumbar spine;
- Risk factors for the onset of LBP are generally physical impairments or training methods-related factors that could be partly modified and managed in clinical practice;
- Scarcity and methodological weakness of the available studies invite to conduct further research about actual prevalence and incidence as well as risk factors for LBP among runners;
- LBP may be better defined as Running Related Disorder instead of Running Related Injury.

## Background

Running is one of the most practiced sports in the adult population worldwide, due to the sustainable cost of technical materials and its great beneficial impacts on health [1–11]. The benefits of running include weight control and prevention of chronic health disorders, such as the cardiovascular diseases, resulting in a general reduction of mortality risk [1–6]. The health benefits associated with running are well-documented, nevertheless the attention to lifestyle, diet, fitness and competitive athletics promoted by media in the last decade, have led to a drastic increase of the levels of physical activity and interest in both competitive and recreational running, even in subjects without an appropriate knowledge on training methodology [3–8]. Although evidence suggests that running is one of the most effective ways to achieve a good state of health and fitness [9], recent studies indicate that it also involves a relatively high risk of associated injury [10, 11]. Several authors have reported that 11–85% of recreational runners have at least one Running Related Injuries (RRIs) each year [10], resulting in a reduction or interruption of training in 30–90% of runners [11–13]. Acute RRIs are rare, almost 80% of RRIs are due to overuse, resulting from an imbalance between the resistance capacity of connective tissue and the biomechanical load of running [14, 15]. The prevalence rate of RRIs among middle and long-distance runners has been reported to range between 19 and 92% [2, 16–20]. However, the discrepancies among studies limit the comparison of data due to the divergences in the type of runners analyzed, follow-up provided, study design, etiology and definition of RRIs [1, 2, 14–25]. In 2015, Yamato et al. [20] defined the RRIs as musculoskeletal pain or physical complaint of the lower limbs or of the back/trunk due to running, causing a total restriction or interruption of running for at least seven or more days and requiring therapeutic assistance [20]. Currently a definition of RRIs is not yet fully share [20], this is reflected in the difficulty of analyzing the studies about of RRIs [18]. RRIs therefore primarily affect joints of the lower limb, pelvis and lumbar spine [18, 25, 26], causing painful muscles, tendons and joints, often resulting in low back pain (LBP) [14–26]. It is frequent in clinical practice [27–33], that patients contact physical therapists for consultancy on LBP which represents a common complaint of athletes [27–33]. In the 90% of the cases, LBP is defined as non-specific, because the patho-anatomical musculoskeletal causes are not clearly identifiable [34]. LBP is one of the most common health problems in the world, that 80% of adults experience at some point in their life [35, 36]. Despite many published studies on the prevalence and incidence of LBP, there is not a clear consensus regarding its actual epidemiologic impact [37–40]. Indeed, some evidence reported a point prevalence estimate of LBP that ranged from 1 to 58% (mean 18.1%) [39, 40]. One-year and lifetime’s prevalence of LBP in the general worldwide population, ranged between 0.8–82.5% (mean 38,1%) and 11–84% (mean 47,1%), respectively [39, 40]. Similarly, regarding a population of athletes [41], the percentage values of the prevalence of LBP remains wide, namely 1–94% in the lifetime (highest prevalence in rowing and cross-country skiing) [41], and 18–65% for the point prevalence (lowest prevalence in basketball and highest prevalence in rowing) [41].

As seen in the general population, a big amount of athletes also experiences LBP [41–48]. Moreover, athletes of particular sport disciplines such as ski, rowing, golf, volleyball, track and fields, swimming or gymnastics are at greater risk of suffering from LBP than non-athletes population [33, 41, 43–48]. The incidence rates of low back pain in athletes have been reported up to 30% depending on the specific sport they are involved in [49]. However different authors describe also a great variability in prevalence rates, that have been reported in a range from 66% [50, 51] to 88.5%, respectively in young athletes and in elite athletes [52]. The incidence rate constitutes the frequency of new events of a medical disorder in the studied population considered at risk, calculated in a given period of time [53]. On the other hand, the prevalence proportion is the part (in percentage) of a defined population affected by a particular medical disorder at a given point in time, or over a specified period of time [53].

Despite several studies about the prevalence and incidence of LBP in general population and sports are retrievable [35–41], it seems that this topic has not been clearly investigated in the runners. Researches are mainly focused on RRIs in general but there are not Systematic Reviews (SRs) specifically addressing prevalence, incidence and risk factors for LBP in runners [11, 18]. Moreover, earlier literature of LBP has been addressed to a wide range of sports or athletes [31, 54] and no conclusive data were published peculiarly on the LBP among a specific population of runners. For this reason, the aims of this systematic review (SR) were to investigate among runners: 1) the prevalence and the incidence of LBP; and 2) specific risk factors for the onset of LBP.

## Methods

### Study design and protocol

The Preferred Reporting Items for Systematic Reviews and Meta-Analyses (PRISMA) protocol was used to design the present SR [55]. This SR has been registered in PROSPERO database (number CRD42018102001).

### Search strategy

An electronic literature search was conducted in the following databases from their inception to 31st of July 2019: PubMed, CINAHL (EBSCO), Google Scholar, Ovid, PsycINFO, PSYNDEX, Embase, SPORTDiscus, Scientific Electronic Library Online (SciELO), Cochrane Library and Web of Science. Research strategies were conducted and designed depending on the specific settings of each database with the supervision of an expert librarian. The research strings were developed according to the PICO model of clinical question (participants, interventions, comparison and outcomes). Free-terms or synonyms (e.g. runners; risk factors; running-related injury), and when possible MeSH (Medical Subject Headings) terms (e.g. low back pain; prevalence; incidence) were used and combined with Boolean operators (AND, OR, NOT). Additionally, a manual research has been conducted through the bibliographies of all the assessed studies to obtain an integrative cross-references full-text selection. A dedicated search strategy was prepared for each database. We have reported the full search strategy for PubMed in Additional file 1.

### Eligibility criteria

All studies were conducted on runners without age limitation. We included any type of study design aiming to investigate prevalence, incidence or risk factors for LBP as RRIs (e.g., cross-sectional, case-control, prospective and retrospective cohort studies). Moreover, single cohort designs were also considered. Runners of any kind of experience or mileage were included, whereas sprinters and track and field athletes were not considered. We defined as RRI any occurrence severe enough to avoid or even restrict the running activity for at least 24 h. We selected studies reporting at least one anatomical area included in LBP definition such as area located below the margin of the 12th rib and above inferior gluteal fold (included: pelvis/pelvis crest, sacrum and gluteus/buttock) [56] and the pathoanatomical cause of the pain cannot be determined [57]. We selected studies published in English or Italian language without limits of date of publication. Descriptive observational designs, such as case report and case series, and any study, which did not meet the inclusion criteria, were excluded.

### Study selection

The selection and data collection process were done by two reviewers (FM and AC) under the supervision of a third author (MT). The whole records were screened by the management software for systematic reviews “Rayyan” (https://rayyan.qcri.org), while references were managed by the “Mendeley” software (https://www.mendeley.com). After the removal of the duplicates, titles and abstracts were screened. Then, full-texts of the identified studies were obtained for further assessment and analyzed independently according to the eligibility criteria by two reviewers (FM and AC). Where appropriate, authors were contacted in order to obtain the full-text.

### Data collection

For each article, the following data was extracted: study design; author, year of publication; the number and characteristics of participants/populations; international definition and/or any diagnostic criteria for LBP; analysis of the variables and the outcome of the studies; study settings/country (e.g., marathon, half-marathon, survey, lab analysis); prevalence and incidence rates; intervention and results; follow-up or study duration; theoretical perspectives on potential risk factors on the onset of LBP: reported risk factors; outcomes and measurements to associate the risks associated with LBP (e.g., relative risk, odds ratio, etc.).

### Quality assessment

The Risk of Bias (RoB) of the included studies is analyzed using the Joanna Briggs Institute Critical Appraisal tools [58] according to the specific study design (e.g., prevalence data, cross-sectional studies, case-control studies, prospective studies). In addition, as prevalence data may be sourced from several numbers of study designs, a critical appraisal checklist specifically for prevalence studies were used. Two independent researchers (FM, LS) evaluated the RoB. The score of RoB was not adopted as criteria to include/exclude studies in this review.

### Agreement

Cohen’s Kappa (K) was used to assess the interrater agreement between the two authors (FM, AC) for full-text selection (K = 0.78; 0.61–0.80 IC 95%). Cohens’ K was interpreted according to Altman’s definition: k < 0.20 poor, 0.20 < k < 0.40 fair, 0.41 < k < 0.60 moderate, 0.61 < k < 0.80 good, 0.81 < k < 1.00 excellent [59].

### Data analysis

We reported the data related to the prevalence, incidence and risk factors for LBP from each study. When needed, we estimated data on prevalence, incidence and risk factors using available data of the included studies. We reported the prevalence and incidence percentage in table form.

## Results

### Study selection process

**Fig. 1:**
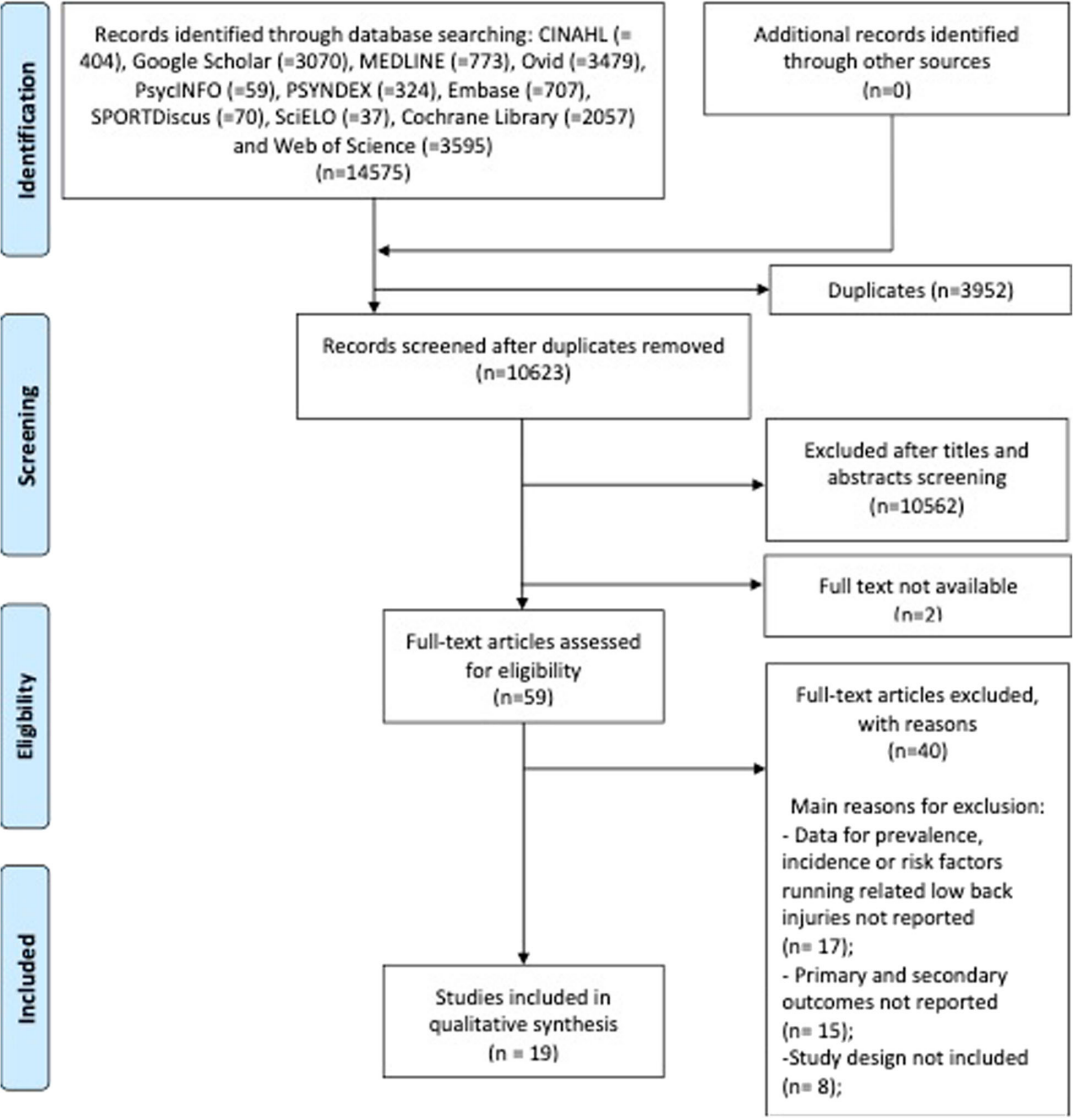
PRISMA 2009 flow diagram

Electronic database searches and the identification of additional references yielded 14,575 records, including 3952 duplicates that were removed. After screening titles and abstracts, 10,564 (including 2 full-text not available) records were excluded. Then, 59 potentially relevant studies were considered eligible for full-text assessment, resulting in 19 included studies in this SR for quality assessment, data extraction and analysis. The selection process is described in Fig. 1 according to the PRISMA Statement [55]. Reasons for exclusions are reported in Table 1.

**Table 1:** Studies Exclusion

First Author, Year	Journal, Title	Reasons For Exclusion
Aggrawal ND, 1979	Br J Sports Med A Study of changes in the spine in weight-lifters and other athletes	Data for prevalence, incidence or risk factors running related low back injuries are not reported This study evaluates the spine complaints in weight-lifters and track and field athletes
Bertelsen ML, 2017 [24]	Scand J Med Sci Sports A framework for the etiology of running-related injuries	Study design is not included
Brill PA, 1995	Sports Med The influence of running patterns on running injuries.	Study design is not included
Buist I, 2010	Am J Sports Med Predictors of running-related injuries in novice runners enrolled in a systematic training program: a prospective cohort study	Primary and secondary outcomes are not evaluated
Burrows M, 2003	Br J Sports Med Physiological factors associated with low bone mineral density in female endurance runners	Primary and secondary outcomes are not evaluated This study analyzes the BMD (bone mass index) of several body segments after physical exercises
Cai C, 2015	J Orthop Sports Phys Ther Low Back and Lower Limb Muscle Performance in Male and Female Recreational Runners with Chronic Low Back Pain	Primary and secondary outcomes not evaluated This study evaluates some physical test such as muscular strength and length
Cole AJ, 1995	J Back Musculoskelet Rehabil Spine injuries in runners: A functional approach	Data for prevalence, incidence or risk factors running related low back injuries are not reported Study design is not relevant
Damsted C, 2019	J Orthop Sports Phys Ther Preparing for half-marathon: The association between changes in weekly running distance and running-related injuries – does it matter how the running is scheduled?	Primary and secondary outcomes are not evaluated. This study evaluates only running-related injuries localized in the lower limb
Fokkema T, 2018	J Sci Med Sport Prognosis and prognostic factors of running-related injuries in novice runners: a prospective cohort study	Data for prevalence, incidence or risk factors running related low back injuries are not reported
Franke TPC, 2019 [5]	J Orthop Sports Phys Ther Running Themselves Into the Ground? Incidence, Prevalence, and Impact of Injury and Illness in Runners Preparing for a Half or Full Marathon	Data for prevalence, incidence or risk factors running related low back injuries are not reported This study grouped the data for head, spine and trunk
Fredericson M, 2007	Sports Med Epidemiology and aetiology of marathon running injuries	Study design is not included
Garbutt G, 1990 [60]	Med Sci Sports Exerc Running speed and spinal shrinkage in runners with and without low back pain	Data for prevalence, incidence or risk factors running related low back injuries are not reported This study analyzes the spinal shrinkage in runners. The authors consider LBP as an independent of the shrinkage induced by running
Hamill J, 2009	Res Sports Med Lower extremity joint stiffness in runners with low back pain	Primary and secondary outcomes are not evaluated. This study evaluates the joint stiffness of hip, knee and ankle in runners with current LBP, resolved LBP and controls
Hespanhol Junior LC, 2016	Scand J Med Sci Sports Health and economic burden of running-related injuries in runners training for an event: a prospective cohort study	Data for prevalence, incidence or risk factors running related low back injuries are not reported
Jacobs S, 1986	Am J Sports Med Injuries to runners: a study of entrants to a 10,000-m race.	Primary and secondary outcomes are not evaluated
Kemler E, 2018 [12]	Phys Sportsmed The relationship between the use of running applications and running-related injuries	Data for prevalence, incidence or risk factors running related low back injuries are not reported
Kluitenberg B, 2013	BMC Public Health The NLstart2run study: health effects of a running promotion program in novice runners, design of a prospective cohort study	Study design is not included
Kluitenberg B, 2016	J Sci Med Sport The NLstart2run study: training-related factors associated with running-related injuries in novice runners	Primary and secondary outcomes are not evaluated. This study analyzes the risk factors for running-related injury without referring to specific anatomical sites for each participant
Lee SP, 2018	Phys Ther Sport Adaptations of lumbar biomechanics after four weeks of running training with minimalist footwear and technique guidance: Implications for running-related lower back pain	Primary and secondary outcomes are not evaluated The authors report only that incorporating minimalist footwear and technique coaching into a runners’ training may induce changes in lumbar biomechanics associated with reduced risk of running related LBP, without any statistical analysis
Lewis G, 2000	ISMJThe etiology and clinical features of low back pain in distance runners: a review	Study design is not relevant
Linton L, 2018	J Sci Med Sport Running with injury: a study of UK novice and recreational runners and factors associated with running related injury	Data for prevalence, incidence or risk factors running related low back injuries are not reported
Lopes AD, 2011	J Physiother Musculoskeletal pain is prevalent among recreational runners who are about to compete: an observational study of 1049 runners	Data for prevalence, incidence or risk factors running related low back injuries are not reported This study evaluated the general spine complaints
Nielsen RO, 2019	BMJ Open The Garmin-RUNSAFE Running Health Study on the aetiology of runningrelated injuries: rationale and design of an 18-month prospective cohort study including runners worldwide	Study design is not included
Nielsen RO, 2013 [61]	Int J Phys Ther Classifying running-related injuries based upon etiology, with emphasis on volume and pace	Study design is not included
Noormohammadpour P, 2015	Eur Spine J Low back pain status of female university students in relation to different sport activities	Data for prevalence, incidence or risk factors running related low back injuries are not reported This study evaluates the LBP status in 9 sports, but not among runner
Ogon M, 1999	Foot Ankle Int Does arch height affect impact loading at the lower back level in running?	Primary and secondary outcomes are not evaluated
Oliveira RR, 2017	Int J Sports Phys Ther There are no biomechanical differences between runners classified by the functional movement screen	Primary and secondary outcomes are not evaluated This study evaluates the timing of TrA (transversus abdominis muscle) activation and the sit and reach test such as possible factors for LBP development
Preece SJ, 2016	Gait Posture How do elite endurance runners alter movements of the spine and pelvis as running speed increases?	Primary and secondary outcomes are not evaluated This study analyzes some cinematics parameters of the spine and pelvis without any consideration for LBP
Sado N, 2017	Sports Biomech The three-dimensional kinetic behaviour of the pelvic rotation in maximal sprint running	Primary and secondary outcomes are not evaluated This study analyzes the lumbosacral cinematic to improve the sprint performance in running
Schafer WE, 1985	Stress Health Life changes, stress, injury and illness in adult runners	Data for prevalence, incidence or risk factors running related low back injuries are not reported
Scheer BV, 2011 [62]	Clin J Sport Med Al Andalus Ultra Trail: an observation of medical interventions during a 219-km, 5-day ultramarathon stage race	Data for prevalence, incidence or risk factors running related low back injuries are not reported
Seay JF, 2014	Eur J Sport Sci Trunk bend and twist coordination is affected by low back pain status during running	Data for prevalence, incidence or risk factors running related low back injuries are not reported This study analyzes the differences in trunk sagittal kinematics between 3 groups of runners, with current LBP, resolved LBP or controls
Smits DW, 2018	Res Sports Med Validity of injury self-reports by novice runners: comparison with reports by sports medicine physicians	Data for prevalence, incidence or risk factors running related low back injuries are not reported This study examines the criterion validity of self-reported running related injuries, compared to an injury consultation by a sport medicine physician
Sugisaki N, 2011	Int J Sport Health Sci The Relationship between 30-m Sprint Running Time and Muscle Cross-sectional Areas of the Psoas Major and Lower Limb Muscles in Male College Short and Middle Distance Runners	Primary and secondary outcomes are not evaluated
Tam N, 2018	J Sports Sci Bone health in elite Kenyan runners	Primary and secondary outcomes are not evaluated
Tauton JE, 2002	Br J Sports Med A retrospective case-control analysis of 2002 running injuries	Data for prevalence, incidence or risk factors running related low back injuries are not reported This study evaluates the running-related injury in athletes of different sports such as cycling, swimming, weight-lifting, etc. who referred to had an injury during running activity
Villavicencio AT, 2006	Neurosurg Focus Back and neck pain in triathletes	Data for prevalence, incidence or risk factors running related low back injuries are not reported The study population is triathlon athletes
Wen DY, 2007	Curr Sports Med Rep Risk Factors for Overuse Injuries in Runners	Study design is not included
Winter SC, 2018	J Phy Fit Treatment & Sports Centre of Mass Acceleration-Derived Variables Detects Differences between Runners of Different Abilities and Fatigue-Related Changes during a Long Distance Over ground Run	Primary and secondary outcomes are not evaluated. This study evaluates the differences in running movements using a wireless accelerometers
Winter SC, 2019	Res Sports Med Overuse injuries in runners of different abilities-a one-year prospective study.	Data for prevalence, incidence or risk factors running related low back injuries are not reported This study provides the total amount of injuries for the groups of running level. The single anatomical site of injury for each runner was not included

### Characteristics of the included studies

The 19 included studies were: cross-sectional (*n* = 6) [63–68]; retrospective (*n* = 3) [69–71]; and prospective (*n* = 10) [72–81]. They were all published in English, starting from 1981 [69] to 2019 [66, 80]. Overall, follow-ups or time duration of these studies ranged from 6 weeks [76] to 2 years [79], while sample sizes varied from a minimum of 4059 to a maximum of 4380 participants [66]. The characteristics of included studies are reported in Table 2.

**Table 2:** Characteristics of the included studies

General informations (Author, years, study design, country)	Title of the study (Journal)	Aims of the study	Population	Incidence or Prevalence	RRI definition	RUNNER definition	LBP (definition, characteristics, anatomical area)	Outcomes and outcome measures
**Bach KD,** **1985** [72] **Prospective Cohort study** **USA**	A comparison of muscular tightness in runners and nonrunners and the relation of muscular tightness to low back pain in runners. (The Journal Of Orthopaedic And Sports Physical Therapy)	To compare muscular tightness at the hip between runners and nonrunners, and to determine if there is a relation between muscular tightness and low back pain in runners.	*N* = 45 Age = 18–43 years M = 28 (19.4 years) F = 17 (25.7 years) LBP = 10 (M = 7 F = 3). Running experience > 1 years	I = 22% (1-year incidence)	Injury severe enough to temporarily give up the running activity.	Runners was defined according to the following criteria: individuals who run 18 or more miles per week, who run on a regular basis, and who have engaged in running for a minimum of 1 year	Low Back Pain	Goniometric range of motion measurements of three hip movements, abduction, flexion with the knee extended, and extension, were taken on two subject populations, runners, and non-runners, in order to determine tightness of the hip adductor, extensor, and flexor muscles, respectively.
**Besomi M,** **2019** [66] **Cross-sectional study** **Chile**	Training volume and previous injury as associated factors for Running-Related Injuries by race distance: A Cross-Sectional Study. (Journal of Human Sport & Exercise)	To determine the relationship between weekly pre-competition running volume and the presence of running-related injuries (RRIs) by race distance.	*N* = 4380 Age = 36 10 km = 1316 21 km = 2168 42 km = 896 LBP = 31 (point prevalence); 307 (1-year overall prevalence; 77 = 10 km; 163 = 21 km; 67 = 42 km) Running experience = < 1 year = 704; 1–4 years = 2226; > 4 years = 1450.	*P* = 13.5% (1-year prevalence); 0.7%(point prevalence)	A running-related injury was defined as “any injury to muscles, tendons, joints and/or bones caused by running. The injury had to be severe enough to cause or be expected to cause a reduction in distance, speed, duration, or frequency of running for at least 7 days. Conditions such as muscle soreness, blisters, and muscle cramps were not considered as injuries”.	Participants was defined as runners if have competed in one of the three SM running distances (10–21-42 km)	Lower Back	
**Buist I,** **2008** [73] **Prospective cohort study** **Netherlands**	Incidence and risk factors of running-related injuries during preparation for a 4-mile recreational running event. (British Journal of Sports Medicine)	The purpose of this study is to determine the incidence of RRI and to identify sex-specific predictors of RRI among a group of novice and recreational runners training during an 8-week period for a 4-mile running event.	*N* = 629 M = 207 F = 422 Age = 43.7 years Running experience = novice runners, runners with previous experience who have taken up running again or runners who were already engaged in regular running. LBP = 31	I = 4.8% (8 weeks)	A running-related injury was defined as any musculoskeletal pain of the lower limb or back causing a restriction in running (mileage, pace or duration) for at least 1 day.	The participants had to categorize themselves as novice runners, runners with previous experience who have taken up running again or runners who were already engaged in regular running. . The training program for novice runners started with ten 1-min repetitions of running alternated by 1 min of walking. The training program for experienced runners started with 30 min of continuous running. The exposure time of running in the training program for novice and recreational runners varied, respectively, between 10 and 40 and 20–60 min per training.	Back Lower Back	
**Chang WL,** **2012** [67] **Cross-sectional study** **Taiwan**	Running injuries and associated factors in participants of ing taipei marathon. (Physical Therapy in Sports)	To investigate the distribution of lower extremity running injuries and their associated factors.	*N* = 893 M = 714 (80%) F = 179 (20%) Age = 20–50 y full marathon group (38.8 11.6 years) 10 km group (33.6 9.8 years) Full marathon = 127 (14.3%); Half marathon = 337 (37.7%);10 km = 429 (47.9%) Running experience: < 1 year = 179; 1-5 years = 435; 5-10 years = 130; > 10 years = 146 LBP = 29; 8 (Full marathon); 11 (Half marathon); 10 (10 km)	*P* = 3.2% (lifetime prevalence)	Questionnaire did not specifically ask the participants to identify if they currently had any symptoms. We would not know how many of the runners only had previous injuries or they also suffered from current injuries. The severity of the running symptoms was not defined in the questionnaire.	Regular running was defined as a minimum of 30 min running at least twice a week.	Lower Back Pain	
**Clement DB,** **1981** [69] **Retrospective survey** **Canada**	A survey of overuse running injuries. (The Physician and Sportsmedicine)	To give an accurate indication of age and sex distributions, training mileage, etiological factors, and the incidence of specific disorders to physicians in sportsmedicine clinics.	*N* = 1650 M = 987 (59.8%) F = 663 (40.2%) Age = 28 years Running experience = recreational runners LBP = 68 (M = 36 F = 32); Nonspecific lower back pain = 54 M = 27 F = 27; Sciatica = 10 M = 10 F = 3; Spondylolysis = 3 M = 2 F = 1; Spondylolisthesis = 1 F = 1;	I = 3.7% (2 years)	Physician diagnosis of RRI	To be regarded as a runner, a patient had to be running at least 2 miles (3 km) three days a week at the time of injury.	Lower back injuries: Nonspecific lower back pain; Sciatica; Spondylolysis; Spondylolisthesis	
**Dallinga J,** **2019** [80] **Prospective cohort study** **Netherlands**	Injury incidence and risk factors: a cohort study of 706 8-km or 16-km recreational runners. (BMJ Open Sport & Exercise Medicine)	To report (1) the injury incidence in recreational runners in preparation for a 8-km or 16-km running event and (2) which factors were associated with an increased injury risk.	*N* = 706 M = 375 F = 331 Age = 43.9 years Running experience = novice and recreational runners LBP = 13 8 km =128 16 km = 521 both distances = 29	I = 1.9% (protocol-event incidence, 12 weeks)	Every physical complaint that caused at least 1 week of training loss.	Participants was defined as runners if have competed at least in one of the two distances of the run (8-16 km).	Lower Back	OSTRC and questionnaire of running training and injury related to running
**Ellapen TJ,** **2013** [70] **Retrospective descriptive study** **Africa**	Common running musculoskeletal injuries among recreational half-marathon runners in kwazulu-natal. (The South African Journal of Sports Medicine)	To document the prevalence and nature of running-related musculoskeletal injuries among recreational half-marathon runners over a 12-month period (1 July 2011–31 June 2012).	*N* = 200 M = 120 F = 80 Age = 43.6 years Running experience = cohort regularly participated in half-marathons (21.1 km), with an average road-running history of 12.2 years. LBP = 28.	*P = 14% (overall 1-year prevalence) *P = 9% male *P = 10% female (* the data of prevalence are related to lower back/hip)	A sensation of distress or agony, and which prevented them from physical activity for a minimum of 24 h	To be regarded as a runners, participants have to had run at least an half marathon (21.1 km)	Lower Back/Hip	
**Kluitenberg B,** **2015** [76] **Prospective cohort study** **Netherlands**	The Nlstart2run Study: Incidence and risk factors of Running-Related Injuries in novice runners. (The Scandinavian Journal of Medicine & Science in Sports)	The purposes of the study were to assess the incidence of RRIs and to identify risk factors for RRIs in a large group of novice runners. In total,1696runnersofa6-weeksupervised“StarttoRun” program were included in the NLstart2run study.	*N* = 1696 M = 364 F = 1332 Age = 43.3 years Running experience = novice runners LBP = 6	I = 0.3%	RRI was defined as a musculoskeletal complaint of the lower extremity or back that the participant attributed to running and hampered running ability for three consecutive training sessions at the same body part. Muscle soreness and blisters were not registered.		Pelvis/Sacrum/Buttock	
**Lysholm J,** **1987** [74] **Prospective cohort study** **Sweden**	Injuries in runners. (The American Journal of Sports Medicine)	To study injury-provoking factors in training and competition, and to compare differences in injury pattern between different groups runners.	*N* = 60 M = 39 F = 11 Age = Sprinters (20.6 ± 3.8), middle-distance runners (18.6 ± 2.4), and long-distance/marathon runners (34.5 ± 7.4) Running experience = 4 years (sprinters) 3 years (middle distance) 5 years (long distance/marathon) LBP = 3	I = 5%	Any injuries that markedly hampered training or competition for at least 1 week were noted.	The participants have a previous experience of running training (7 h per week or more).	Low Back Pain	
**Marti B,** **1988** [68] **Cross-sectional survey** **Switzerland**	On the epidemiology of running injuries, the 1984 bern grand-prix study. (The American Journal of Sports Medicine)	- How frequent are jogging injuries in a representative population comparing all participants in a popular running event, and how often do such injuries lead to medical consultation and absence from work? - Is the incidence of jogging injuries related to behavior and/or characteristics of runners (training mileage, type of running shoes, age, number of years of training, etc.)? - What are the site and nature of the most common injuries? - Is there a relation between specific runner characteristics and specific running injuries?	*N* = 4358 M = 4358 Age = 35.0 years LBP = 30	*P* = 0.7% overall 1-years LBP - Grade III injuries (2.2% lower back; 0.6% pelvic crest; 0.9% buttock)	Runners were asked to classify jogging injuries that had occurred during the previous 12 months according to their effect on running. Grade I injuries involved maintenance of full training activity in spite of symptoms; Grade II, a reduction of training activity, and Grade III, full training interruption, defined as involuntary complete interruption of running of at least two weeks’ duration.		Lower Back Buttock Pelvic Crest	
**Malliaropoulos N,** **2015** [64] **Cross-sectional study** **Greece**	Prevalence of injury in ultra trail running. (Human Movement)	The purpose of the study was to try to determine the prevalence of lower extremity and lower back musculoskeletal injuries in ultra-trail runners by considering injuries and related symptoms. Additionally, the predicting factors associated with these injuries were investigated in order to aid in the prevention and rehabilitation of trail running injuries.	*N* = 40 ultra-trail runners M = 36 F = 4 Age = 38.4 years (22–59) Running experience = Level A = 13; Level B = 27; < 6 years = 21; > 6 years = 19 LBP = 17	*P* = 20.2% (lifetime)	If symptoms were severe enough to forgo training for at least 1 day or causing them to quit a race.	According to the International Trail running Association, trail running takes place on various natural terrain (mountain, desert, or forest) while minimizing running on paved or asphalt surfaces (no more than 20% of the total distance in competition). It can involve uphill, downhill, and horizontal trails and is similar in duration to an ultra-marathon, which is considered any race beyond the marathon distance of 42.195 km.	Lower Back Lower Back Pain	
**Messier SP,** **2018** [79] **Prospective cohort study** **USA**	A 2-year prospective cohort study of overuse running injuries, the runners and injury longitudinal study (TRAILS). (The American Journal of Sports Medicine)	To determine the risk factors that differentiate recreational runners who remain uninjured from those diagnosed with an overuse running injury during a 2-year observational period.	*N* = 300 M = 172 F = 128 Age = 41.15 Running experience = 11.2% LBP = 18	I = 6% (2 years)	Overuse running injuries were graded with the method defined by Marti et al.: grade 1, maintained full activity in spite of symptoms; grade 2, reduced weekly mileage; and grade 3, interrupted all training for at least 2 weeks	Participants was defined as runners if they run a minimum of 5 miles per week.	Back	-Exercise Self-efficacy Scale (0, lowest self-efficacy; 100, highest self-efficacy)—which assesses beliefs in the ability to continue to run at one’s training pace for periods of 1 to 8 weeks24. − 12-Item Short Form Health Survey (SF-12) healthrelated quality-of-life survey (0, low; 100, high)—which measures perceived health (mental subscale) and functioning (physical subscale). -Satisfaction With Life Scale (5, extremely dissatisfied; 35, highly satisfied)—which assesses global judgment of life satisfaction. -Positive and Negative Affect Scale (PANAS) (10, very slightly; 50, extremely for each scale). -State-Trait Anxiety Inventory–S Scale (20, not at all; 80, very much)—which asks participants to report how they feel right now. -Visual analog scale for pain (0, no pain; 10, extreme pain).
**Rasmussen CH,** **2013** [71] **Retrospective cohort study** **Denmark**	Weekly running volume and risk of running-related injuries among marathon runners. (The International Journal of Sports Physical Therapy)	The purpose of this study was to investigate if the risk of injury declines with increasing weekly running volume before a marathon race	*N* = 662 M = 535 F = 127 Age = 41.4 Running experience = marathon runners: < 2 years = 49; 2–5 years = 262; > 5 years = 351; LBP = 3	I = 0.5% point incidence	The running-related injury definition was modified based on the injury definition used by Macera et al.; a running-related injury was defined as an injury to muscles, tendons, joints and/or bones caused by running; The injury had to be severe enough to cause or be expected to cause a reduction in distance, speed, duration, or frequency of running for at least 14 days. Conditions like muscle soreness, blisters, and muscle cramps were not considered as injuries.	Completion of the H.C. Andersen marathon	Lower Back	
**Tauton JE,** **2003** [75] **Prospective cohort study** **Canada**	A prospective study of running injuries: the vancouver sun run “in training” clinics. (British Journal of Sports Medicine)	To determine the injury pattern in a sample of the “In Training” clinics during their 13 weeks program, and identify associated risk factors for injury.	*N* = 840 M = 205 F = 635 Age = 30–56 years Running experience = novice runners LBP = 37	I = 1.6% Low back = 7% (4 men), =5% (10 women) -Hip/pelvis: =7% (4 men), =10% (19 women)		-The novice group is primarily sedentary and deconditioned people interested in establishing a running program probably to improve health and fitness. The program for this group incorporates run/walk repeats that eventually lead to a continuous running session in the 12th week. -The intermediate program is designed for people who have completed the novice walk/run program and would like to increase their running endurance and intensity in a safe and effective way. Hill training, interval, and fartlek sessions are implemented.	Low Back	
**Teixeira RN,** **2016** [65] **Cross-sectional study** **Brazil**	Prevalence of musculoskeletal pain in marathon runners who compete at the elite level. (The International Journal of Sports Physical Therapy)	The purpose of this research was to assess the prevalence, location and intensity of running related musculoskeletal pain over the previous 12 months in marathon runners who compete at the elite level and to verify whether certain training characteristics are associated with musculoskeletal pain.	*N* = 199 M = 164 F = 35 Age = 34 (30–39) Running experience = marathon runners at elite level, on average of 11 years; LBP = 28 (Lumbar Spine 20; Pelvic/Sacral/Gluteus 8)	P = 14% (1-year prevalence)		Runners who compete at the elite level, defined as those competing at international and/or national level; Their training is characterized by a high training volume, with weekly training loads of up to 160 km/week.	Lumbar Spine Pelvic/Sacral/Gluteus	VAS
**van der Worp MP,** **2016** [77] **Prospective cohort study** **Netherlands**	The 5- or 10-km Marikenloop run: a prospective study of the etiology of running-related injuries in women. (Journal of Orthopaedic & Sports Physical Therapy)	To determine the incidence and characteristics (site and recurrence) of running-related injuries and to identify specific risk factors for running-related injuries.	*N* = 373 (5 km = 189; 10 km = 184) F = 373 Age = 37.55 Running experience = novice runners LBP = 10 (4 Lower Back; 6 Buttock)	I = 2.7%	Defined as running-related pain of the lower back and/or the lower extremity that restricted running for at least 1 day.	Adult women (aged ≥18 years) who had signed up for the ‘Marikenloop 2013’ running event were eligible for inclusion. The ‘Marikenloop’ is a run over 5- or 10 km in Nijmegen, the Netherlands, and is a female-only event.	Lower Back Buttock	
**Von Rosen P,** **2017** [78] **Prospective cohort study** **Sweden**	Etiology of running-related injuries in women. (The International Journal of Sports Physical Therapy)	The aims of this study were to describe the injury prevalence/incidence, severity grade, injury location, risk factors and the prevalence of illness in running (RU), orienteering (OR) and cross-country skiing athletes (CR).	*N* = 189 F = 189 Age = range 18–24 Running experience = elite athletes LBP = 5	I = 2.8%	As any physical complaint that affected participation in normal training or competition, led to reduced training volume, experience of pain or reduced performance in sports; * A substantial injury was defined as an injury leading to moderate or severe reductions in training volume, or moderate or severe reduction in performance, or complete inability to participate in sports; * A new injury was categorized as a recurrent or a non-recurrent injury, based on if the injury occurred in the same body site as the previous injury within the last year.		Low Back Pain Lower Back	OSTRC
**Walter SD,** **1989** [81] **Prospective cohort study** **USA**	The Ontario Cohort Study of Running-Related Injuries. (Archives of Internal Medicine)	The purpose of this study was to investigate the incidence and causes of running injuries.	*N* = 1288 M = 985 F = 303 Age = 41.4 Running experience= LBP = 23 new injuries; 56 old injuries	I = 1.8% (point incidence) *P* = 4.3% (1-year prevalence)		All registered entrants to these events: 16-km (10-mile) race, 4-km (2.5-mile) fun run in St Catharines. 22.4-km (14mile) run and a four-member 5.6-km (3.5-mile) team relay in Burling ton were included.	Back Back Injuries	
**Woolf SK,** **2002** [63] **Cross-sectional survey** **USA**	The Cooper River Bridge Run of low back pain in runners and walkers. (Journal of the Southern Orthopaedic Association)	The purpose of this study was to investigate the incidence, prevalence, and possible risk factors for LBP among a group of runners and walkers.	*N* = 436 M = 227 F = 209 Age = 36.45 years Running experience = any kind of runner from novice to athletes LBP = 59	*P* = 13.6% (point prevalence)			Low Back Pain LBP	

### Risk of Bias of the included studies

Details of the RoB of the included studies are presented in Tables 3, 4, 5 and 6. Most items of all RoB assessment tools used for the quality assessment were rated as low risk. For all the studies addressing prevalence data regardless of the study design [63–65, 67, 68, 70, 81], the items rated as unclear RoB were related to the sampling methods in 3 studies [63, 65, 70], while in one study the items rated as high risk [68]. More in depth in one study [68], another two items were rated as high risk, one regarding the reliability of the condition measurement and one regarding the validity of identification of the condition. For cross-sectional studies, the majority of studies had low and, less commonly, unclear RoB [63–68]. However, among them, in the study of Marti et al. [68], the item related to the criteria for inclusion was rated as high risk, likewise the item about the reliability of the condition measurement in the study of Chang et al. [67]. For retrospective studies [69–71] there was a low RoB across all the studies, apart from comparability of groups, matching of cases and controls, adoption of the same criteria for identification of case and controls and methods to measure the exposure in 3 studies [69–71], which were all rated as not applicable. Finally, for prospective studies [72–81], in 6 studies [71, 73–76, 79] items related to the similarity/recruitment of groups, methods of exposure were rated as not applicable. Also were judged as not applicable the items related to the time of follow-up and loss to follow-up in the study of Back et al. [72]. Moreover, in 3 studies [72, 74, 78] the item about strategies to address incomplete follow up was evaluated as not applicable, whereas the remaining items were commonly judged as low RoB.

**Table 3:** Case Control Critical Appraisal

	Clement DB. 1981 [69]	Ellapen TJ. 2013 [70]	Rasmussen CH. 2013 [71]
Were the groups comparable other than the presence of disease in cases or the absence of disease in controls?	Not applicable	Not applicable	Not applicable
Were cases and controls matched appropriately?	Not applicable	Not applicable	Not applicable
Were the same criteria used for identification of cases and controls?	Not applicable	Not applicable	Not applicable
Was exposure measured in a standard, valid and reliable way?	Unclear	Yes	Yes
Was exposure measured in the same way for cases and controls?	Not applicable	Not applicable	Not applicable
Were confounding factors identified?	Yes	Yes	Yes
Were strategies to deal with confounding factors stated?	Yes	Yes	Yes
Were outcomes assessed in a standard, valid and reliable way for cases and controls?	Yes	Yes	Yes
Was the exposure period of interest long enough to be meaningful?	Yes	Yes	Yes
Was appropriate statistical analysis used?	Unclear	Yes	Yes

**Table 4:** Cohort Critical appraisal

	Dallinga J. 2019 [80]	Bach DK. 1985 [72]	Buist I. 2008 [73]	Lysholm J. 1987 [74]	Tauton JE. 2003 [75]	Van der Worp MP. 2016 [77]	Messier SP. 2018 [79]	Kluitenberg B. 2015 [76]	Von Rosen P. 2017 [78]	Walter SD. 1989 [81]
Were the two groups similar and recruited from the same population?	Yes	Yes	Not applicable	Yes	Not applicable	Not applicable	Yes	Not applicable	Not applicable	Not applicable
Were the exposures measured similarly to assign people to both exposed and unexposed groups?	Yes	Yes	Not applicable	Yes	Not applicable	Not applicable	Yes	Not applicable	Not applicable	Not applicable
Was the exposure measured in a valid and reliable way?	Yes	Yes	Yes	Yes	Unclear	Unclear	Yes	Unclear	Unclear	Unclear
Were confounding factors identified?	Yes	Unclear	Yes	Unclear	Yes	Yes	Yes	Yes	Yes	Yes
Were strategies to deal with confounding factors stated?	Unclear	Unclear	Yes	Unclear	Yes	Yes	Yes	Yes	Yes	Yes
Were the groups/participants free of the outcome at the start of the study (or at the moment of exposure)?	Unclear	Yes	Unclear	Yes	Unclear	Unclear	Yes	Yes	Unclear	Unclear
Were the outcomes measured in a valid and reliable way?	Yes	Yes	Yes	Unclear	Yes	Yes	Yes	Yes	Yes	Yes
Was the follow up time reported and sufficient to be long enough for outcomes to occur?	Yes	Not applicable	Unclear	Yes	Yes	Yes	Yes	Unclear	Yes	Yes
Was follow up complete, and if not, were the reasons to loss to follow up described and explored?	Yes	Not applicable	Yes	Yes	Yes	Yes	Yes	Yes	Yes	Yes
Were strategies to address incomplete follow up utilized?	Yes	Not applicable	Yes	Not applicable	Unclear	No	Yes	Yes	Not applicable	Yes
Was appropriate statistical analysis used?	Yes	Yes	Yes	Yes	Yes	Yes	Yes	Yes	Yes	Yes

**Table 5:** Cross Sectional Critical Appraisal

	Chang WL. 2012 [67]	Malliaropoulos N. 2015 [64]	Marti B. 1988 [68]	Woolf S. 2002 [63]	Teixeira RN. 2016 [65]	Besomi M., 2019 [66]
Were the criteria for inclusion in the sample clearly defined?	Yes	Yes	No	No	Yes	Yes
Were the study subjects and the setting described in detail?	Yes	Yes	Yes	Yes	Yes	Yes
Was the exposure measured in a valid and reliable way?	Yes	Yes	Yes	Yes	Yes	Yes
Were objective, standard criteria used for measurement of the condition?	No	Yes	Yes	Yes	Yes	Yes
Were confounding factors identified?	Unclear	Yes	Yes	Yes	Yes	Yes
Were strategies to deal with confounding factors stated?	Unclear	Yes	Yes	Unclear	Yes	Yes
Were the outcomes measured in a valid and reliable way?	Unclear	Yes	Yes	Unclear	Yes	Yes
Was appropriate statistical analysis used?	Yes	Yes	Yes	Yes	Yes	Yes

**Table 6:** Prevalence Studies Critical Appraisal

	Chang WL. 2012 [67]	Marti B. 1988 [68]	Ellapen TJ. 2013 [70]	Malliaropoulos N. 2015 [64]	Walter SD. 1989 [81]	Teixeira RN. 2016 [65]	Woolf S. 2002 [63]	Besomi M. 2019 [66]
Was the sample frame appropriate to address the target population?	Yes	Yes	Yes	Unclear	Yes	Yes	Yes	Yes
Were study participants sampled in an appropriate way?	Yes	No	Unclear	Yes	Yes	Unclear	Unclear	Yes
Was the sample size adequate?	Yes	Yes	Yes	Unclear	Yes	Yes	Yes	Yes
Were the study subjects and the setting described in detail?	Yes	Yes	Yes	Yes	Yes	Yes	Yes	Yes
Was the data analysis conducted with sufficient coverage of the identified sample?	Yes	Yes	Yes	Yes	Yes	Yes	Yes	Yes
Were valid methods used for the identification of the condition?	Yes	No	Yes	Yes	Yes	Yes	Unclear	Yes
Was the condition measured in a standard, reliable way for all participants?	Yes	No	Yes	Yes	Yes	Yes	Yes	Yes
Was there appropriate statistical analysis?	Yes	Yes	Yes	Yes	Yes	Yes	Yes	Yes
Was the response rate adequate, and if not, was the low response rate managed appropriately?	Yes	Yes	Yes	Yes	Yes	Yes	Unclear	Yes

### Summary of findings

Results about prevalence and incidence are reported in Table 7.

**Table 7:** Results about prevalence and incidence of LBP

Author, year	Study design	Incidence				Prevalence			
		Lifetime	1 year	Point	Other	Lifetime	1 year	Point	Other
Bach DK, 1985 [72]	Prospective Cohort		22%						
Besomi M, 2019 [66]	Cross Sectional Survey						13.5%	0.7%	
Buist I, 2008 [73]	Prospective Cohort				4.8% (protocol event: 8 weeks)				
Chang WL, 2012 [67]	Cross Sectional Survey					3.2%			
Clement DB, 1981 [69]	Retrospective Survey				3.7% (2 years)				
Dallinga J, 2019 [80]	Prospective Cohort				1.9% (protocol event: 12 weeks)				
Ellapen TJ, 2013 [70]	Retrospective Descriptive						14%		
Kluitenberg B, 2015 [76]	Prospective Cohort				0.3% (protocol event: 6 weeks)				
Lysholm J, 1987 [74]	Prospective Cohort		5%						
Marti B, 1988 [68]	Cross Sectional Survey						0.7%		
Malliaropoulos N, 2015 [64]	Cross Sectional					20.2%			
Messier SP, 2018 [79]	Prospective Cohort				6% (2 years)				
Rasmussen CH, 2013 [71]	Retrospective Cohort			0.5%					
Tauton JE, 2003 [75]	Prospective Cohort				1.6% (protocol event: 13 weeks)				
Teixeira RN, 2016 [65]	Cross Sectional						14%		
Van der Worp MP, 2016 [77]	Prospective Cohort				2.7% (12 weeks)				
Von Rosen P, 2017 [78]	Prospective Cohort		2.8%						
Walter SD, 1989 [81]	Prospective Cohort			1.8%			4.3%		
Woolf SK, 2002 [63]	Cross Sectional Survey							13.6%	

### Prevalence of LBP

Eight [63–68, 70, 81] of the 19 included studies addressed prevalence of LBP among runners. Six were cross-sectional studies [63–68], one was a retrospective study [70] and one was a prospective study [81]. The range of point prevalence ranged from a minimum of 0,7% [66] at a maximum of 13.6% [63] and lifetime prevalence ranged from a minimum of 3.2% [67] a maximum of 20.2% [64]. Point prevalence values, 13.6 and 0.7% respectively, were reported in two studies [58, 64]. Five studies reported values of 1-year prevalence [65, 66, 68, 70, 81], ranged from 14% [65, 70] to 0.7% [68], and those about lifetime prevalence were two [63, 64], 3.2 and 20.2% respectively. Only 1 study [66] addressed data for point and 1-year prevalence, with values of 0.7 and 13.5% respectively [66]. Also in the cross-sectional survey study of Woolf et al. [63], the point prevalence of LBP in runners was reported, and it was equal to 13.6%. The study of Marti et al. [68] reported a 1-year prevalence of LBP of 0.7%, but this value was calculated in a sample of all male runners, and it was referred only to the Grade III injuries (defined as full training involuntary interruption of running for at least 2 weeks duration). In the cross-sectional study of Teixeira et al. [65] 1-year prevalence of LBP (including pain in the lumbar spine and pain in pelvic/sacral/gluteus regions) among elite marathon runners was 14%. In the retrospective descriptive study of Ellapen et al. [70] the 1-year prevalence of lower back (including hip) among recreational half-marathon runners was 14% (mean value; 13% for men, 15% for women). In the only prospective cohort study, Walter et al. [81], the 1-year prevalence of LBP among 1288 runners was 4.3%. The highest lifetime prevalence rate of LBP was reported to be 20.2% in the cross-sectional study of Malliaropoulos et al. [64] in a sample of 40 ultra-trail runners. Furthermore, in another cross sectional study [67], the LBP lifetime prevalence was 3.2% [67], in a sample of 893 subjects of which 80% of male runners [67].

### Incidence of LBP

Twelve [69, 71–81] of the 19 included studies addressed incidence of LBP among runners. Ten were prospective studies [72–81], and two were a retrospective study [69, 71]. Overall, the incidence of LBP among runners ranged from a minimum of 0.35% (in 6 weeks) [76] and maximum value of 22% (in 1-year) [72]. The highest incidence rate of LBP was reported as equal to 22% (7 male; 3 female) in the prospective study of Bach et al. [72] in a sample of 45 runners.

The minimal rate of incidence, below 1%, was found in the studies by Kluitenberg et al. [76] and Rasmussen et al. [69] with values of 0.35 and 0.5%, respectively.

Furthermore, overall low incidence values, beneath 5%, were found in other six studies [69, 73, 75, 77, 78, 80, 81]. Among them, a value of 1.6% (in 13 weeks) was found in the prospective study of Tauton et al. [73] for the distribution of injuries in the lower back. A similar value (1.8%) was found in Walter et al. [81]. In a more recent prospective cohort study of Dallinga et al. [80] an incidence rate of 1.9% (in 12 weeks) was found in a sample of recreational runners, during the training period for a running event. More in depth, the analysis of Van Der Worp et al. [77] showed a rate of 2.7% (in 12 weeks) in a sample of adult women runners. Moreover, the prospective cohort study of Von Rosen et al. [78] reported the incidence of injuries in the lower back of 2.8% of all injuries recorded between young female runners (mean age 17 years). Lastly, in the study of Buist et al. [73], a value of 4.8% (in 8 weeks) was found among a sample of novice runners, in runners with previous experience who have started running again and runners engaged in regular running [73]..

In the remaining two prospective cohort studies [74, 79], the incidence rate of LBP was found to be slightly higher. Indeed, Lysholm et al. [74] reported a 1-years incidence equal to 5% among a small sample of 39 runners and in the recent study of Messier et al. [79] the incidence (in 2 years) of LBP among runners was 6%, considering the anatomical sites of back and pelvis. In the end, the retrospective analysis of Clement et al. [69] among 1650 runners revealed similar findings: the 2 years-incidence of injuries localized in the lower back was 3.7% (3.3% for men and 4.3% for women).

#### Risk factors for LBP

The risk factors for the onset of LBP are reported in Table 8. Four studies [63, 64, 69, 70] addressed specific risk factors for LBP in runners. Two of them were retrospective studies [69, 70] and two were cross sectional studies [63, 64]. The retrospective analysis of Clement et al. [69] indicated as possible risk factors for the development of non-specific back pain in runners leg-length discrepancy, poor hamstrings flexibility and poor back flexibility [69]. However, the authors did not specify the strength of the associations with LBP and the values of statistical significance. In another retrospective study [70] on recreational runners’ tightness of hip flexors and hip flexion angle measured both with the Thomas test (a clinical and physical test used to measure the flexibility of the hip flexors, which includes the iliopsoas muscle group, the rectus femoris, pectineus, gracillis as well as the tensor fascia latae and the sartorius [82] and goniometer were defined as potential intrinsic factors predisposing to lower back/hip injuries. Indeed, the hip flexion angles of female runners who have suffered lower back/hip musculoskeletal injuries were significantly greater, than those of their not-injured counterparts (*p* < 0.01) [70]. Risk factors are listed in Table 8.

**Table 8:** Risk Factors for the onset LBP

Author	Risk Factors For LBP	P -Value	Odds Ratio
Clement DB, 1981 [69]	- Leg-length discrepancy - Reduced hamstrings flexibility - Reduced back flexibility	/ / /	
Ellapen TJ, 2013 [70]	- Hip flexion angles (female) -(Thomas Test + goniometer)	p < 0.01	†3.0488
Malliaropoulos N, 2015 [64]	- > than 6 years of experience in running	P = 0.012	†5.4857
Woolf SK, 2002 [63]	- Not equal wear of heels^1^ - BMI ≥ 24^1^ - Not performing Weekly aerobics activity^1^ - Not Play in contact sports regularly^1^ (i.e. football, soccer, basketball, wrestling, boxing, rugby - Not using orthotics + not equal wear of heels^1^ - Outside pattern of wear^a,1^ - Running without Inside pattern of wear^2^ - Higher Physical height; - Flexibility exercises routine for a longer time before working out^2^ - Not doing Traditional aerobics activity^2^	p = 0.034 p < 0.01 p < 0.05 p < 0.04 p = 0.011 *p* = 0.013 *p* ≤ 0.02 p ≤ 0.02 p ≤ 0.05 p < 0.05	‡1.263 (female ^BMI) ‡1.122 (male ^BMI)

* higher credits as a sum of sex and age of the runner, difficulty level of previous races – positive height difference, the vertical climb index, and the distance in km – and performance.1 Previous LBP; a Subgroup of runners without insert; 2 Current LBP; †value calculated by authors using data from the full-text;‡ value reported directly from the full-text

Moreover, the cross-sectional study of Malliaropoulos et al. [64] highlighted that having more than 6 years of experience of running could represent a predicting factor for getting injured in the lower back (*p* = 0.012) [64].

Lastly, Woolf et al. [63], in a cross sectional study conducted on a wide sample of runners, showed that runners who have previously suffered LBP, have reported greater shoe wear on either the inside or outside. Conversely, an equal shoe wear was less likely to relate a previous history of LBP (*p* = 0.034) [63].

In the same study [63], a previous history of LBP was reported by runners who did not use orthotics (such as insert, insole, heel, foot-bed, etc.) (*p* = 0.011), by who had a body mass index higher than 24 (p < 0.01) and by who did not perform weekly traditional aerobics activity (*p* < 0.05).

Moreover, again in the study of Woolf et al. [63], runners who did not regularly play contact sports (e.g., football, soccer, basketball, wrestling, boxing, rugby) were more likely (*p* < 0.04) to have suffered LBP than those who do [63]. Current LBP was reported by high stature (p < =0.02) runners and by who perform a long time flexibility exercises routine before the training (p < =0.05) [63].

## Discussion

The aim of this SR was to investigate the prevalence and incidence of LBP and to identify risk factors for the onset of LBP among runners. To the best of the authors’ knowledge, this is the first SR addressing these outcomes in this specific population.

### Prevalence and incidence of LBP

Despite running is one of the most practiced sports worldwide, and the prevalence rate of RRI is well documented in scientific literature [1–11], prevalence and incidence of LBP among runners are still unclear. The relatively low number of studies that we were able to include in the present review confirms the scarcity of literature on this topic.

Overall, the findings of this SR revealed that LBP prevalence and incidence among runners, compared to the general population [35–40], were low. In detail, according to the results, within the most represented sports population among the analyzed studies (i.e. 20–50 years of age), running does not appear to be related to higher rates of incidence and prevalence of LBP, if compared to the general population presenting same age [40, 83, 84].

Indeed, within the general population, the point prevalence estimate of LBP was described in a range of 1–58% (mean 18.1%) [39, 40], while in our review the point prevalence was 0.7–13.6%, however retrievable only in two studies. The one-year and lifetime prevalence of LBP, calculated in the worldwide population, ranged between 0.8 and 82.5% (mean 38.1%) and 11–84% (mean 47.2%), respectively [39, 40]. Conversely, in our review, the one-year and lifetime prevalence ranged between 0.7 and 14% [65, 66, 68, 70, 81] and 3.2% [67] and 20.2% [64], respectively. The same considerations may be made for the incidence, indeed the one-year incidence in the general population was 36% [39], while data emerging from our SR indicate that 1-year incidence reported a range from 2.8% [78] to 22% [72].

Moreover, it should be noted that the results of two studies reporting high prevalence (20.2% lifetime) [64], and high incidence (22% 1-year) [72], is probably depending from the very small [64, 72] and the specific sample of 40 ultra-trail runners (that face with races taking place on mountain, desert, or forest and it includes uphill, downhill and is similar in duration to an ultra-marathon, that is beyond the distance of a regular marathon of 42.195 km) [64].

Regarding the mileage, it is worth pointing out that LBP prevalence in runners seems to be somehow independent of the running distance. In Besomi et al. [66] the largest sample (4380) within studies included in our SR, prevalence was assessed on a race of three difference distances (10, 21 and 42 km). The rate of prevalence in the 42 km-runners was similar (7.5%) to the rate among the 21 km-runners, (7.5%).

Moreover, the findings of this SR revealed that the LBP prevalence and incidence in runners seem to be less relevant compared with the benchmarks of RRI in literature [2, 10, 11, 16–20, 61, 62, 79–81, 85, 86]. Indeed, the RRIs affecting lower limbs seem to have much greater prevalence rates, reporting a range of value of 28–42% for the knee (e.g., patellar tendinopathy, iliotibial band syndrome, patellofemoral pain syndrome), and of 14–38% for the ankle (e.g., ankle sprain, Achilles tendinopathy, plantar fasciopathy) [16–20, 61, 62, 79–81, 85–87].

Although prevalence and incidence of LBP appear low if compared to the general population, this conclusion should be taken cautiously. Indeed, out of the scarcity of the available studies, there are many points in the included studies that weaken the generalizability of this statement.

Further studies are needed in order to extend the results of this systematic review to the older adult population, because LBP incidence and prevalence values increase with age [83, 84].

Transversely to all the included studies, participants were heterogeneous for individual characteristics (age, sex), training level and previous injuries. Therefore, it is reasonable that various samples of populations (ex. young elite athletes or middle-aged recreational runners) may led to different prevalence or incidence rates. Furthermore, as reported in some prospective studies, not all the participants were exposed to the same running or training methods. In the cross-sectional survey study of Woolf et al. [63] for example, the rate of LBP point-prevalence, was calculated not only within experienced runners, but also between novice runners. Instead, in the study of Marti et al. [64] the 1-year prevalence of LBP, 0.7%, was estimated in a wide sample, 4358 runners, but constituted of only male runners; In the cross-sectional study of Teixeira et al. [65] which is the only one to report the International Association for the Study of Pain (IASP) definition of pain [86], the prevalence of LBP was calculated among elite marathon runners who compete at international and/or national level and perform high volume of training, up to 160 km/week. In the cross-sectional study of Chang et al. [67] in a sample of 893 runners (mostly composed of male) although the lifetime prevalence rate was low, 3.2%, runners were not specifically asked if they had the symptom at the time of completing the questionnaire. Concerning the incidence, in the prospective study of Bach et al. [72] the highest rate of LBP (22%; 7 males, 3 females) was found within a small sample of 45 runners. In the two prospective cohort studies [77, 78], the rate of incidence was assessed in samples made up exclusively of female runners and four studies evaluated incidence rates of LBP in only novice runners [75–77, 79]. Clement et al. [69] was the only study that used the term “Non-specific lower back pain”, as reported by the most recent literature [57, 88] and only seven among the included studies [63, 64, 66, 69, 72, 74, 78], to define an injury affecting the lumbar spine, adopted specific terms such as low/lower back pain, LBP, Non-specific lower back pain.

### Risk factors

Only four studies addressed specific risk factors for the onset of LBP among runners [63, 64, 69, 70] and great caution is required for translating their results to general practice being those studies two retrospective studies [69, 70] and the two cross-sectional studies [63, 64], which do not represent the most reliable study design to assess risk factors.

According to the Comprehensive Model for Injury Causation [60] and the Conceptual Model for the Determinants of RRIs [89], intrinsic and extrinsic factors are responsible for the increase of running injury risk. Intrinsic factors are hardly or not modifiable; they include sex [60, 89], age [60, 89], BMI [60, 89], history of previous injury [60, 89], physical fitness and psychological factor have been found to predispose runners to injury [60, 89]. Otherwise, extrinsic factors are modifiable, and comprise training volume or other characteristics, as sport equipment and training environment, which rise the runner’s susceptibility to injury [60, 89]. Intrinsic risk factors proposed for the onset of LBP among runners included: BMI ≥24 [63]; higher physical height [63]; tightness of hip flexors (measured by Thomas Test) [70] and hip flexion angles (only in female and measured by goniometer) [70]; but, as referred by the authors, there is no strong literature to explain this two last finding [70]. Moreover, the identification by Clement et al. [67] of physical impairments, like reduced hamstring or back flexibility and leg length discrepancy, was not supported by statistical evaluation. Notably, if the runners are compared to non-runners, these seem to present a significantly lower degree of hip flexion with the knee extended, indicating a tightness of hamstrings (*p* < 0.001). Nonetheless, no correlation was found between muscular tightness in runners and the incidence of LBP [72]. Due to the scarcity of available studies and the clinical impression that muscles tightness could be a risk factor for RRIs and LBP, this topic should be investigated in large samples using prospective design.

The main extrinsic risk factors for the onset of LBP among runners were: high competitive level [64]; more than 6 years of experience in running [64]; some patterns of shoes’ wear [63] and do not performing weekly aerobics activity [63].

Also in this case the findings extracted from the two selected studies [63, 64] cannot be directly translated to the daily practice, but could only serve as possible additional elements to support the clinician in the interpretation of the athlete’s condition. Indeed, the exposure to a single risk factor is often insufficient to produce an overuse injury: the RRI is the result of a number of superposing factors (like training increase, muscular impairments, unsuitable equipment, etc.) [66].

### Consistency

There is a need of a standard and internationally acceptable definitions for LBP and a clearer definition and terminology of RRI. RRI is defined as an overuse injury due to an unbalance between the resistance capacity of connective tissue and the biomechanical solicitations of running [14, 15]. Therefore, here the meaning of “injury” differs from usual meaning which is related to an acute trauma and, in a clinical perspective, very rare among runners [3, 14, 15].

In our view, a more suitable word may be “Disorder” (Running Related Disorders - RRDs) that better describes multifactorial conditions which include, beside structural aspect, also psychosocial elements often present in nonspecific painful disorders like LBP [90–93].

Our SR confirmed, also for running, the findings of a recent SR [33] which concluded that the evidence about prevalence of LBP in athletes of some popular sports are scarce and derived from studies not of good methodological quality. This SR showed a quite high LBP prevalence among athletes, but this finding was relative to a wider sample of sports including volleyball, track and fields, swimming, golf, ski, gymnastics and rowing [33, 43–48], not specifically including running.

### Clinical implications

Despite a significant correlation between spinal shrinkage, running speed and distance covered exists, it is not correlated to the onset or presence of LBP [94]. Moreover, some studies suggested that running could have an anabolic role towards the intervertebral disc [95–98], among them Belavý et al. [97] reported that long-distance runners and joggers showed better hydration and glycosaminoglycan levels than the non-athletic individuals.

These findings, together with the low level of incidence and prevalence of LBP among runners, cautiously invite thinking running as a protective factor from LBP and to consider of prescribing running as a preventive exercise for LBP.

Although the data available on risk factors are weak and not conclusive, nevertheless most of proposed running related risk factors were modifiable by specific intervention and adapted training and they should be taken into consideration by physical therapists and trainers.

### Implications for research

More high-quality studies that analyse the prevalence and incidence of LBP in runners are needed before drawing strong and definitive conclusions. The actual prevalence and incidence of LBP in runners should be investigated by large cohort studies, adopting better definition of the clinical symptoms, rather than just pain distribution in anatomical districts. Moreover, a consensus on the definition of RRIs that consider the inclusion of psychosocial aspect and widens the usual pathoanatomic approach is advisable due the characteristics of conditions like LBP.

Risk factors should be assessed by methodologically sound prospective studies on more homogeneous populations (in terms of demographic characteristics, training level of participants, gender, age, etc.). As reported by our results, running seems to represent a sport without an increased rate of LBP: data of LBP prevalence and incidence among runners are lower than those found in other sports [33, 41]. However, caution is required when assuming that run could be a good practice in order to prevent LBP. Our findings mostly derive from novice runners or recreational runners, so it would be pretentious to apply in other sports and in elite/professional contexts.

### Perspective

Running is one of the most practiced sports [1–11] and although evidence suggests that is one of the most effective ways to achieve a good state of health [9], recent studies indicate that it also involves a relatively high risk of injuries [10, 11]. Currently a definition of RRIs is not yet fully share, this is reflected in the difficulty of analyzing the studies about of RRIs [18]. RRIs primarily affect joints of the lower limb and lumbar spine [18, 25, 26, 99], causing painful muscles, tendons and joints, also resulting in LBP [14–26], but despite several studies about the prevalence and incidence of LBP in sports are retrievable [35–40], it seems that this topic has not been clearly investigated in the runners. Therefore, the aetiology, the prevalence and the incidence of LBP, likewise the RRI, have been reviewed. Specifically, it is important to consider how often the effectiveness of a given RRI prevention intervention is dependent on an easy modification of etiologic factors, and on and their consistency with a biologically plausible causal mechanism [24]. Therefore, the investigation of how different factors affect the lumbar spine, in terms of structure-specific load and/or loadability, and the dose-response relationship between running participation and injury risk [24]. These considerations allow researchers to move beyond traditional risk factor identification. Just so, research findings could be reliable, not only in terms of the observed cause-effect association but also translatable in clinical practice [24].

Moreover, although the encouraging results, they are limited to the population most represented among the studies analysed (20–50 years of age), from this perspective, running activity could be used as a strategy to maintain a healthy lifestyle in the adult population, as indicated by the World Health Organization guidelines (WHO) [100].

### Limitations

This SR has several limits. Studies written in languages other than English or Italian were excluded and, due to the heterogeneity of the included studies were not possible to perform a meta-analysis.

We have not included sprinter runners in the research strategies, as keyword. Moreover, we have adopted a strict topographical definition of LBP [56], while in other epidemiological studies the authors referred as a LBP a generic “back pain”, which could involve even the thoracic region [34, 40, 83, 84, 101]. Moreover, a homogeneous definition of LBP was not adopted in all studies, populations investigated were different and prevalence, incidence or risk factors for the onset of LBP are investigated by questionnaires that are exposed to recall bias.

Furthermore there is a high risk of selection bias in the studies, in that persons with LBP may not be able to run, increasing the rate of prevalence of LBP in general population.

Lastly, being unavailable a specific and validated assessment tool for retrospective studies, for the assessment of the methodological quality of the included studies was adopted the tool designed for case-control studies.

Finally, it would be also necessary to tighten up the definitions of both incidence and prevalence rates [53], which are sometimes confused or inverted, and therefore create difficulties in the interpretation of data.

## Conclusion

Despite the small number of included studies, the heterogeneity of the samples investigated and of running modalities did not allow to gain conclusive results, the prevalence and incidence of LBP among runners appear to be low if compared to the general population and to other RRIs. Most of the physical and training-related risk factors for the onset of LBP, even based on weak evidence, are potentially modifiable by a careful intervention of the clinician and should be considered when LBP prevention is sought.

## Supplementary information

**Additional file 1.** Database search Strategy.

## Data Availability

All data generated or analyzed during this study are included in this published study. Other information of this study are available from the corresponding author on reasonable request.

## Abbreviations

LBP: Low Back Pain
SR: Systematic Review
RoB: Risk of Bias
RRIs: Running Related Injuries
RRDs: Running Related Disorders
PROSPERO: Prospective Register of Systematic Reviews
WHO: World Health Organization
