# Supplementary material for: Prevalence and incidence of low back pain among runners: a systematic review
Source: BMC Musculoskelet Disord. 2020 Jun 3;21:343. doi: 10.1186/s12891-020-03357-4 (PMC7271446; doi:10.1186/s12891-020-03357-4)
Supplement: Supplementary file 1 — Additional file 1. Database search Strategy. [file 12891_2020_3357_MOESM1_ESM.docx]

**Supplementary Materials**

**Additional file 1. Database search Strategy**

| **QUERY FOR PUBMED**  "running"[MeSH Terms] OR "Track and Field"[Mesh] OR (runn[tiab] OR runnability[tiab] OR runnable[tiab] OR runnabledbs[tiab] OR runnables[tiab] OR runnagarden[tiab] OR runnan[tiab] OR runnanensis[tiab] OR runnard[tiab] OR runne[tiab] OR runnebaum[tiab] OR runneberg[tiab] OR runned[tiab] OR runnegar[tiab] OR runnel[tiab] OR runneled[tiab] OR runneling[tiab] OR runnelled[tiab] OR runnelling[tiab] OR runnells[tiab] OR runnels[tiab] OR runnemede[tiab] OR runner[tiab] OR runner'[tiab] OR runner's[tiab] OR runneri[tiab] OR runnering[tiab] OR runnerless[tiab] OR runnerlike[tiab] OR runners[tiab] OR runners'[tiab] OR runneth[tiab] OR runngin[tiab] OR runnig[tiab] OR runnin[tiab] OR runnin'[tiab] OR runniness[tiab] OR running[tiab] OR running'[tiab] OR running''[tiab] OR running's[tiab] OR runningback[tiab] OR runningbacks[tiab] OR runningbased[tiab] OR runningcoach[tiab] OR runninghills[tiab] OR runningly[tiab] OR runningmap[tiab] OR runningmel[tiab] OR runnings[tiab] OR runnings'[tiab] OR runningshoe[tiab] OR runningwave[tiab] OR runningwheel[tiab] OR runninr[tiab] OR runnners[tiab] OR runnning[tiab] OR runnstrom[tiab] OR runnstrom's[tiab] OR runnung[tiab] OR runny[tiab] OR runnymede[tiab]) OR (jogg[tiab] OR jogged[tiab] OR jogged'[tiab] OR jogger[tiab] OR jogger's[tiab] OR joggers[tiab] OR joggers'[tiab] OR joggin[tiab] OR joggin'[tiab] OR jogging[tiab] OR jogging'[tiab] OR joggins[tiab] OR joggitis[tiab] OR joggitis'[tiab] OR joggle[tiab] OR joggling[tiab]) OR "Track and Field"[tiab]) AND ("back pain"[All Fields] OR ("low back pain"[MeSH Terms] OR ("low"[All Fields] AND "back"[All Fields] AND "pain"[All Fields]) OR "low back pain"[All Fields]) OR "lumbar pain"[All Fields] OR "backache"[All Fields] OR "lumbago"[All Fields] OR ("low back pain"[MeSH Terms] OR ("low"[All Fields] AND "back"[All Fields] AND "pain"[All Fields]) OR "low back pain"[All Fields] OR ("lower"[All Fields] AND "back"[All Fields] AND "pain"[All Fields]) OR "lower back pain"[All Fields]) AND ") AND ("[All Fields] AND ("musculoskeletal pain"[MeSH Terms] OR ("musculoskeletal"[All Fields] AND "pain"[All Fields]) OR "musculoskeletal pain"[All Fields]) AND "[Mesh] OR "[All Fields] AND ("soft tissue injuries"[MeSH Terms] OR ("soft"[All Fields] AND "tissue"[All Fields] AND "injuries"[All Fields]) OR "soft tissue injuries"[All Fields]) AND "[Mesh] OR "[All Fields] AND ("sprains and strains"[MeSH Terms] OR ("sprains"[All Fields] AND "strains"[All Fields]) OR "sprains and strains"[All Fields]) AND (("running"[MeSH Terms] OR "running"[All Fields]) AND ("injuries"[Subheading] OR "injuries"[All Fields] OR "wounds and injuries"[MeSH Terms] OR ("wounds"[All Fields] AND "injuries"[All Fields]) OR "wounds and injuries"[All Fields])) OR (related[All Fields] AND ("running"[MeSH Terms] OR "running"[All Fields]) AND ("injuries"[Subheading] OR "injuries"[All Fields] OR "wounds and injuries"[MeSH Terms] OR ("wounds"[All Fields] AND "injuries"[All Fields]) OR "wounds and injuries"[All Fields])) AND ") AND ("[All Fields] AND ("risk factors"[MeSH Terms] OR ("risk"[All Fields] AND "factors"[All Fields]) OR "risk factors"[All Fields]) AND "[Mesh] OR "[All Fields] AND ("etiology"[Subheading] OR "etiology"[All Fields] OR "causality"[MeSH Terms] OR "causality"[All Fields]) AND (Determinant[All Fields] AND tiab[All Fields] AND determinants[All Fields] AND tiab[All Fields] AND ("risk"[MeSH Terms] OR "risk"[All Fields]) AND tiab[All Fields] AND ("risk"[MeSH Terms] OR "risk"[All Fields] OR "risks"[All Fields]) AND tiab[All Fields] AND ("etiology"[Subheading] OR "etiology"[All Fields] OR "causality"[MeSH Terms] OR "causality"[All Fields]) AND tiab[All Fields]) AND (("cohort studies"[MeSH Terms] OR ("cohort"[All Fields] AND "studies"[All Fields]) OR "cohort studies"[All Fields]) AND ("medical subject headings"[MeSH Terms] OR ("medical"[All Fields] AND "subject"[All Fields] AND "headings"[All Fields]) OR "medical subject headings"[All Fields] OR "mesh"[All Fields])) OR Cohort[tiab] OR cohorts[tiab] OR longitudinal[tiab] OR follow-up[tiab] OR followup[All Fields] OR ("observational study"[Publication Type] OR "observational studies as topic"[MeSH Terms] OR "observational study"[All Fields])) |
| --- |
